# Why It Cannot Represent Trained Nurses

**Published:** 1916-09-09

**Authors:** 


### THE NATIONAL POOR-LAW OFFICERS' ASSOCIATION.

### WHY IT CANNOT REPRESENT TRAINED NURSES.

Some readers of The Hospital or of the *Poor-Law Officers' Journal* may have formed the conclusion that a serious crisis, inimical to the interests of Poor-Law institution nurses, has arisen. They may also have been led to believe, as has been stated, that we are unfairly prejudiced against and antagonistic to Poor-Law work in general and the National Poor-Law Officers' Association and its *Journal* in particular. Neither conclusion is in accordance with facts.

The "critical" developments which are now taking place in the nursing world are those promulgated by the College of Nursing, Limited. The College, as is well known, is seeking powers to form a Nurses Register and to place the training and control of nurses under a General Nursing Council. In connection with this scheme we have heard no suggestion of any intention to discriminate between nurses trained in hospitals supported by voluntary contributions and those trained in the rate-supported Poor-Law infirmaries, or between trained nurses working in the one or the other class of institution. There is nowhere, so far as we know, any desire to see perpetuated any distinction of the Poor-Law trained nurses, who were fully represented at the St. Thomas's Hospital meeting. The first Register of Nurses is open to all who hold a certificate of three years' reasonably satisfactory training, and even to some who do not. Unless, and until, it is clear that the nurses trained in any particular class of institution are being unfairly treated there is no occasion to regard their position as "critical" or urgently to adopt means to protect their interests.

If any "crisis" there were it would be one concerning Poor-Law nurses as nurses and not as Poor-Law officers. Therefore, whilst we question the present necessity for any special organisation of Poor-Law nurses with a view to the protection of their class interests, we have no doubt whatever that, in any case, the National Poor-Law Officers' Association is not the body to interfere upon their behalf in a nursing question. The Association does not represent, and has never represented, the trained Poor-Law nurses; most certainly not those of the separate infirmaries, the great Poor-Law training-schools. The action of the Association in connection with the Poor-Law Officers' Superannuation Act is a notorious instance of its neglect of the interests and needs of nurses and of some other female officers. The Act was framed for the especial benefit of Poor-Law clerks, relieving officers, and workhouse masters; so useless and unfair to nurses were its provisions that, at first compulsorily included, they had in justice to be given the right of refusing its doubtful benefits—a right which the vast majority exercise.

Now, tardily, apparently without any desire or request upon their part, the Poor-Law Officers' Association seeks to pose as champion of the nurses' cause. It wishes to "voice their needs," to organise them, and to "interest them in the question of the College of Nursing." What are the needs which call for this intervention? What has this body of Poor-Law officers to do with the College of Nursing? Other Poor-Law workers—medical men, solicitors, chaplains—do not discuss professional questions or require introduction to professional developments through this Association: the medical officers have their own Association, the medical superintendents of the separate infirmaries their own Society. Surely it is a roundabout and undignified way for nurses who happen to be working in these particular institutions to approach a professional nursing question through a body which can only emphasise the Poor-Law attributes of their work! These nurses are perforce connected with a system confessedly effete and unpopular. In the opinion of many competent judges the time has long since come when the interests of public assistance to the sick would be best served by disseverance from public assistance to the destitute and from all Poor-Law traditions. Mr. Tom Percival, President of the National Poor-Law Officers' Association, in a letter to the Editor, which follows, asks "on what grounds we state so emphatically that this Association stands for the maintenance of the *status quo*" in Poor-Law matters? He does not deny that it does so stand, he pleads that economic considerations after the war must "put back all developments for at least a generation." The *Journal* of his Association says (on August 25) that we are "obviously aware that the Association is making headway and that there is very little or no probability that the Poor-Law hospitals or infirmaries will be dissevered from the present system": the association of ideas is to be observed. The *Journal* also pleads that nurses should cleave to the Association and "attach themselves to methods of real progress and not to fanciful theories which may, or may not, take effect in the lifetime of the next generation."

Mr. Percival hopes that "when we appreciate the reasons which dictate" the campaign of the Association we shall understand its aims. The conclusion is unavoidable that the Association is seeking an opportunity to enlist in its ranks a hitherto indifferent and increasingly important and powerful section of Poor-Law officers. Misled by this ambition, it exaggerates the value of the services which it is capable of rendering for the nurses' benefit, and Mr. Percival speaks of the extent of these prospective services, but does not explain what is the nature of those proffered. We quite appreciate that very valuable services may be rendered by the Association to nurses in their capacity as officers employed by Board of Guardians under the supervision of the Local Government Board. As nurses, who are trained nurses first and Poor-Law servants afterwards, and in purely nursing questions, this Poor-Law Officers' Association is not the proper body to represent them. We do not believe that nurses working in these special institutions are so isolated as to be in time of need without co-operation, support, or advice upon purely professional questions unless under the motherly wing so disinterestedly extended by the Poor-Law Officers' Association.

Tom Percival, President,

National Poor-Law Officers' Association,

